# Abdominal aortic aneurysm events in the women’s health initiative: cohort study

**DOI:** 10.1136/bmj.a1724

**Published:** 2008-10-14

**Authors:** Frank A Lederle, Joseph C Larson, Karen L Margolis, Matthew A Allison, Matthew S Freiberg, Barbara B Cochrane, William F Graettinger, J David Curb

**Affiliations:** 1 VA Medical Center, Minneapolis, MN 55417, USA; 2 Women’s Health Initiative Clinical Coordinating Center, Fred Hutchinson Cancer Research Center, Seattle, WA 98109; 3 HealthPartners Research Foundation, Minneapolis, MN 55440-1524; 4 University of California San Diego, La Jolla, CA 92093-0811; 5 University of Pittsburgh, Pittsburgh, PA 15215; 6 University of Washington School of Nursing, Seattle, WA 98195-7262; 7 University of Nevada School of Medicine, Reno, NV 89557; 8 University of Hawaii School of Medicine, Honolulu, HI 96813

## Abstract

**Objective** To assess the association between potential risk factors and subsequent clinically important abdominal aortic aneurysm events (repairs and ruptures) in women.

**Design** Large prospective observational cohort study with mean follow-up of 7.8 years.

**Setting** 40 clinical centres across the United States.

**Participants** 161 808 postmenopausal women aged 50-79 enrolled in the women’s health initiative.

**Main outcome measures** Association of self reported or measured baseline variables with confirmed abdominal aortic aneurysm events assessed with multiple logistic regression.

**Results** Events occurred in 184 women and were strongly associated with age and smoking. Ever smoking, current smoking, and amount smoked all contributed independent risk. Diabetes showed a negative association (odds ratio 0.29, 95% confidence interval 0.13, 0.68), as did postmenopausal hormone therapy. Positive associations were also seen for height, hypertension, cholesterol lowering treatment, and coronary and peripheral artery disease.

**Conclusions** Our findings confirm the strong positive associations of clinically important abdominal aortic aneurysm with age and smoking in women and the negative association with diabetes previously reported in men.

## Introduction

Aortic aneurysms cause around 15 000 deaths a year in the United States, and 40% of these deaths are in women.^1^ Most result from abdominal aortic aneurysm, which are several times more common in men^2^ ^3^ ^4^ ^5^ ^6^ but are more deadly in women.^7^ ^8^ The aetiology of aortic aneurysm and the reasons for these sex differences remain unknown. Most studies of abdominal aortic aneurysm have focused primarily on men, and little reliable information is available for women. Previous studies have generally included too few women with aneurysms to generate robust multivariable models and, perhaps as a result, have been inconsistent with respect to several factors and unable to determine whether the negative association with diabetes observed in men also occurs in women.^2^ ^3^ ^6^ ^9^ ^10^ ^11^ ^12^

A better understanding of the factors associated with aortic aneurysms in women would allow more accurate assessment of sex differences and might improve diagnostic vigilance and provide insights into pathophysiology. The women’s health initiative was a large complex clinical investigation of strategies for long term prevention of common diseases involving 161 808 women at 40 clinical centres and comprising a set of overlapping clinical trials and an observational study.^13^ ^14^ We assessed the associations between potential risk factors and subsequent clinically important abdominal aortic aneurysm events (rupture and repair) in postmenopausal women.

## Methods

This analysis included all 161 808 women enrolled in the women’s health initiative clinical trial (68 132) or observational study (93 676). Eligible women were postmenopausal, aged 50-79 at entry, had an expected survival of at least three years, and could be expected to adhere to the protocol. Women were enrolled during 1993-8 and followed up until the end of the main study in 2004-5. The study population, recruitment methods, baseline data collection, enrolment, and follow-up procedures have been previously reported.^13^ ^14^ The protocol and consent forms were approved by the institutional review boards of the participating institutions, and written informed consent was obtained from all participants.

Except for the prospective identification of abdominal aortic aneurysm events (described below), the data used in this analysis were obtained at the time of enrolment. Participants were asked if a doctor had ever told them that they had each condition considered, including a previous diagnosis of abdominal or thoracic aortic aneurysm. There was no confirmation of these baseline reports. Height, weight, waist circumference, and blood pressure were measured directly.

For this analysis, we classified hypertension as blood pressure >140/90 or use of anti-hypertensive drugs at entry. Coronary artery disease included history of myocardial infarction, angina, or coronary revascularisation. Cerebrovascular disease included history of stroke, transient ischaemic attack, or carotid revascularisation. Peripheral artery disease included “claudication or peripheral artery disease (poor blood flow to the legs or blocked or narrowed arteries to the legs).” Drugs for high cholesterol included both current and past use. Venous thromboembolism included history of deep venous thrombosis or pulmonary embolus. Chronic obstructive pulmonary disease included emphysema and chronic bronchitis. Non-skin cancer also included melanoma.

An abdominal aortic aneurysm event was recorded when a participant was admitted to hospital during the study period with an aneurysm that was symptomatic or required intervention (such as vascular or surgical procedure), or both, and had a diagnostic or interventional procedure that demonstrated the aneurysm. These events were identified when participants or their proxies completed standardised questionnaires (every six months for participants in the clinical trial and every year for those in the observational study) that asked about all admissions since the last update. A centrally trained local physician reviewed relevant medical records to ensure that the event met the criteria described above. Because a symptomatic abdominal aortic aneurysm nearly always represents rupture, these events should essentially all be ruptures and repairs (either elective or emergent).

We performed multiple logistic regression with SAS version 9.1. Missing responses were treated as “no” for specific diseases, including prevalent aortic aneurysm. We included an indicator variable (not shown) for missing values of “dugs for high cholesterol” because this question was not asked in the early months of the study (nor were cholesterol concentrations measured or women asked about high cholesterol). We excluded participants with missing responses to other variables, including hypertension. The model was adjusted for participation in the clinical trial and assigned treatment. We used Pearson correlation coefficient to evaluate correlations between covariates and ran additional models to evaluate potential interactions between strong predictors.

## Results

At entry into the study, 301 women reported that they had been told by a doctor that they had aortic aneurysm. During follow-up (mean 7.8 years, median 7.9 years), 184 abdominal aortic aneurysm events were reported, 18 in the women who had reported a diagnosis of aortic aneurysm at entry, resulting in 467 women (0.3%) with aortic aneurysm before or during the study. Fourteen of the 184 women who had abdominal aortic aneurysm events died within five days of the event; all 14 deaths were attributed to “other cardiovascular cause,” the category under which deaths related to aneurysm should be coded. Table 1 shows characteristics of the women with and without events[Table tbl1]. Our study population had lower rates of current smoking and most diseases than in women in previous screening studies of abdominal aortic aneurysm.^2^ ^3^ ^9^

**Table 1 tbl1:** Characteristics at time of enrolment of women with and without abdominal aortic aneurysm (AAA) events. Figures are percentages of women unless stated otherwise

Characteristic*	AAA event (n=184)	No event (n=161 624)
Mean (SD) age (years)	67.2 (6.2)	63.2 (7.2)
White	86.4	82.5
Black	8.2	9.0
Mean (SD) height (cm)	162.2 (6.2)	161.8 (6.7)
Mean (SD) weight (kg)	73.0 (17.2)	73.6 (16.9)
Mean (SD) waist circumference (cm)	88.8 (13.9)	86.5 (13.8)
Ever smoked (≥100 cigarettes)	82.6	48.4
Mean (SD) pack years of smokers	44.0 (28.6)	21.0 (22.2)
Current smoker	38.0	6.9
Hypertension	62.5	38.9
Drugs for high cholesterol	27.7	13.3
Coronary artery disease	26.6	6.8
Cerebrovascular disease	9.2	3.2
Peripheral artery disease	12.0	2.0
Venous thromboembolism	3.3	3.9
Diabetes mellitus	4.3	5.9
Chronic obstructive pulmonary disease	12.0	3.5
Non-skin cancer	10.3	9.2
Previous diagnosis of aortic aneurysm	9.8	0.2
Postmenopausal hormone therapy:		
Current	19.6	40.1
Past	18.5	16.1
Alcohol use:		
Never	7.1	10.9
Past drinker	28.3	18.6
<1 drink/week	28.8	32.7
1– <7 drinks/week	20.1	25.5
≥7 drinks/week	15.2	11.6

Table 2 shows multivariable odds ratios for the various factors for abdominal aortic aneurysm events[Table tbl2]. The ratio of events to covariates is higher than the 8:1 needed for robust logistic regression modelling.^15^ After we eliminated waist circumference because of high correlation with weight, the highest remaining correlation (other than those between smoking variables) was 0.26 for height and weight. Women excluded for missing values were otherwise similar to those included for all characteristics examined.

**Table 2 tbl2:** Multivariable model* of factors associated with abdominal aortic aneurysm event

Factor†	Odds ratio (95% CI)
No of women with/without an event‡	164/147 885
Age (per 10 years)	1.77 (1.37 to 2.29)
Black race (*v* white)	0.77 (0.43 to 1.38)
Other race (*v* white)	1.23 (0.63 to 2.39)
Height (per 10 cm)	1.50 (1.15 to 1.96)
Weight (per 10 kg)	0.96 (0.86 to 1.07)
Ever smoked (≥100 cigarettes)	1.94 (1.16 to 3.24)
Current smoker (*v* past smoker)	4.19 (2.93 to 6.01)
Pack years (per 5 pack years)	1.11 (1.08 to 1.14)
Hypertension (>140/90 or medication)	2.14 (1.51 to 3.03)
Drugs for high cholesterol	1.66 (1.15 to 2.39)
Coronary artery disease	2.38 (1.61 to 3.51)
Cerebrovascular disease	1.15 (0.66 to 2.00)
Peripheral artery disease	1.81 (1.05 to 3.11)
Venous thromboembolism	0.58 (0.25 to 1.34)
Diabetes mellitus	0.29 (0.13 to 0.68)
Chronic obstructive pulmonary disease	1.44 (0.88 to 2.37)
Non-skin cancer	0.94 (0.57 to 1.56)
Previous diagnosis of aortic aneurysm	9.00 (4.71 to 17.20)
Current hormone therapy (*v* never)	0.48 (0.31 to 0.73)
Past hormone therapy (*v* never)	0.76 (0.50 to 1.14)
Current alcohol use (*v* never)	0.73 (0.39 to 1.36)
Past alcohol use (*v* never)	0.95 (0.49 to 1.83)

*Model also adjusted for participation in clinical trial and assigned treatment.

†Status at time of enrolment.

‡After exclusion of women with missing values.

Abdominal aortic aneurysm events were strongly associated with age and smoking and, as expected, with previous diagnosis of aortic aneurysm. There were negative associations with diabetes and with baseline use of postmenopausal hormone therapy. Positive associations with abdominal aortic aneurysm events were also seen for height, hypertension, use of cholesterol lowering drugs, and coronary and peripheral artery disease.

There was a significant interaction between smoking and diabetes (P<0.005), such that diabetes amplified the increased risk from smoking of having an event. When we compared current smoking with never smoking in a model that included pack years and all other variables, the odds ratio (95% confidence interval) for abdominal aortic aneurysm events was 8.73 (5.04 to 15.12).

When we used duration of hormone therapy rather than current or past status, the odds ratio for having an abdominal aortic aneurysm event for up to five years’ use was 0.69 (0.46 to 1.04) and for more than five years’ use was 0.52 (0.34 to 0.78).

As shown in table 2, the small group of women who never used alcohol were not at reduced risk of aneurysm events.[Table tbl2] In other models, we separately compared those reporting seven or more alcohol drinks a week, one to fewer than seven drinks a week, or past use with a control group of women who never used alcohol or had less than one drink a week, and found no significant associations of alcohol use with aortic aneurysm events.

## Discussion

In this large cohort of postmenopausal women we have confirmed the strong positive associations of abdominal aortic aneurysm events with age and smoking and the negative association with diabetes, previously reported in men.^2^ ^3^ ^4^ ^5^ ^6^ ^10^

Previous screening studies that used ultrasonography have reported factors associated with abdominal aortic aneurysm in 3000-3500 women each,^2^ ^3^ ^6^ ^9^ but the numbers found to have abdominal aortic aneurysm (1-2%) were too small to generate robust models with more than a few independent variables.^15^ Similarly, a recent report of the Life Line screening programme included 10 012 women but detected only 74 aneurysms.^12^ Previous clinical diagnosis studies from Chicago and northern California identified 109 and 115 abdominal aortic aneurysm events in women, respectively,^10^ ^11^ though only the northern California study provided multivariable models by sex.^11^ Strong associations with abdominal aortic aneurysm in women were found in all these studies for age and smoking and in some for coronary and cerebral vascular disease, and weaker associations were seen in most for height, hypertension, and high cholesterol.

A negative association between abdominal aortic aneurysm and diabetes was described more than a decade ago,^4^ ^16^ and, though confirmed in various studies in men,^5^ ^17^ whether it occurs in women has been uncertain. Several studies found marked negative trends in women,^2^ ^9^ ^11^ but these did not reach significance. The Life Line screening programme did not observe a negative trend,^12^ and the Chicago clinical diagnosis study reported a significant univariable interaction between sex and diabetes, with a positive univariable association for women but a negative trend for men.^10^ In our large study we examined factors associated with abdominal aortic aneurysm in women and found a significant negative association with diabetes. No adequate explanation of the mechanism of this negative association has yet emerged,^18^ but the observation adds to the evidence for a common pathophysiology of abdominal aortic aneurysm in men and women and for a fundamental difference between aneurismal and occlusive vascular disease.^19^

We observed a negative association between postmenopausal hormone therapy and aortic aneurysm, which was significant for use at time of enrolment and for duration of use over five years. There is some precedent in the literature for this association: the northern California study reported a non-significant trend in the same direction,^11^ and a recent review of animal data found evidence for “a possible role for estrogen in protection against abdominal aortic aneurysm.”^8^ On the other hand, 33 of the 184 aneurysm events described in our analysis have been included in previous publications from the women’s health initiative, and these reported hormone therapy effects from the randomised trials that differ from the effects we observed from hormone therapy used before enrolment. Women randomised to hormone therapy had significantly more abdominal aortic aneurysm events than controls in the Estrogen Alone trial (14 *v* 6 cases, hazard ratio 2.4)^20^ and a number similar to controls in the Estrogen plus Progesterone trial (7 *v* 6, hazard ratio 1.1).^21^ Possible sources of discrepancies between baseline and randomised effects of hormone therapy in the women’s health initiative have been reviewed and include both greater duration of treatment and possible confounding by differences in lifestyle in the baseline data.^22^ Further studies will be needed to clarify the effect of hormone therapy on aortic aneurysm.

The small but significant association we found between height and aortic aneurysm has been seen in previous studies in both men and women,^4^ ^5^ ^9^ ^10^ ^11^ even when abdominal aortic aneurysm was defined relative to the suprarenal aorta rather than by unadjusted diameter,^4^ though to our knowledge no explanation for this association has been proposed. Neither our study nor the northern California study^11^ provide support for a recent report of an association between alcohol consumption and abdominal aortic aneurysm.^23^

It remains unclear why prevalence of abdominal aortic aneurysm differs so much by sex, even after multivariable adjustment for known associations. These adjustments might have been incomplete or there might be an as yet undiscovered biological explanation, possibly related to sex steroid hormones. Regardless, the negative association of abdominal aortic aneurysm with diabetes seems to be common to both sexes.

**What is already known on this topic**

Abdominal aortic aneurysm is more common in men but more deadly in women
Most previous studies have included too few women with aortic aneurysm to generate robust multivariable models

**What this study adds**

There are strong positive associations between age and smoking and clinically important abdominal aortic aneurysm in women
The negative association between abdominal aortic aneurysm and diabetes previously reported in men is also seen in women

## Notes

We thank Joann Manson for suggesting this analysis.

The WHI investigators

Program Office: Elizabeth Nabel, Jacques Rossouw, Shari Ludlam, Joan McGowan, Leslie Ford, Nancy Geller (National Heart, Lung, and Blood Institute, Bethesda, Maryland).

Clinical Coordinating Center: Ross Prentice, Garnet Anderson, Andrea LaCroix, Charles L Kooperberg, Ruth E Patterson, Anne McTiernan (Fred Hutchinson Cancer Research Center, Seattle, WA); Evan Stein (Medical Research Labs, Highland Heights, KY); Steven Cummings (University of California at San Francisco, San Francisco, CA).

Clinical Centres: Sylvia Wassertheil-Smoller (Albert Einstein College of Medicine, Bronx, NY); Aleksandar Rajkovic (Baylor College of Medicine, Houston, TX); JoAnn E Manson (Brigham and Women’s Hospital, Harvard Medical School, Boston, MA); Charles B Eaton (Brown University, Providence, RI); Lawrence Phillips (Emory University, Atlanta, GA); Shirley Beresford (Fred Hutchinson Cancer Research Center, Seattle, WA); Lisa Martin (George Washington University Medical Center, Washington, DC); Rowan Chlebowski (Los Angeles Biomedical Research Institute at Harbor-UCLA Medical Center, Torrance, CA); Yvonne Michael (Kaiser Permanente Center for Health Research, Portland, OR); Bette Caan (Kaiser Permanente Division of Research, Oakland, CA); Jane Morley Kotchen (Medical College of Wisconsin, Milwaukee, WI); Barbara V Howard (MedStar Research Institute/Howard University, Washington, DC); Linda Van Horn (Northwestern University, Chicago/Evanston, IL); Henry Black (Rush Medical Center, Chicago, IL); Marcia L Stefanick (Stanford Prevention Research Center, Stanford, CA); Dorothy Lane (State University of New York at Stony Brook, Stony Brook, NY); Rebecca Jackson (Ohio State University, Columbus, OH); Cora E Lewis (University of Alabama at Birmingham, Birmingham, AL); Cynthia A Thomson (University of Arizona, Tucson/Phoenix, AZ); Jean Wactawski-Wende (University at Buffalo, Buffalo, NY); John Robbins (University of California at Davis, Sacramento, CA); F Allan Hubbell (University of California at Irvine, CA); Lauren Nathan (University of California at Los Angeles, Los Angeles, CA); Robert D Langer (University of California at San Diego, LaJolla/Chula Vista, CA); Margery Gass (University of Cincinnati, Cincinnati, OH); Marian Limacher (University of Florida, Gainesville/Jacksonville, FL); J David Curb (University of Hawaii, Honolulu, HI); Robert Wallace (University of Iowa, Iowa City/Davenport, IA); Judith Ockene (University of Massachusetts/Fallon Clinic, Worcester, MA); Norman Lasser (University of Medicine and Dentistry of New Jersey, Newark, NJ); Mary Jo O’Sullivan (University of Miami, Miami, FL); Karen Margolis (University of Minnesota, Minneapolis, MN); Robert Brunner (University of Nevada, Reno, NV); Gerardo Heiss (University of North Carolina, Chapel Hill, NC); Lewis Kuller (University of Pittsburgh, Pittsburgh, PA); Karen C Johnson (University of Tennessee Health Science Center, Memphis, TN); Robert Brzyski (University of Texas Health Science Center, San Antonio, TX); Gloria E Sarto (University of Wisconsin, Madison, WI); Mara Vitolins (Wake Forest University School of Medicine, Winston-Salem, NC); Michael Simon (Wayne State University School of Medicine/Hutzel Hospital, Detroit, MI).

Ethical approval: Not required.

Provenance and peer review: Not commissioned; externally peer reviewed.

## Notes

**Cite this as: ***BMJ* 2008;337:a1724
